# A Decade of GigaScience: Women in Science: Past, Present, and Future

**DOI:** 10.1093/gigascience/giac069

**Published:** 2022-06-29

**Authors:** Lijun Liu, Dragomirka Jovic, Laurie Goodman

**Affiliations:** Lars Bolund Institute of Regenerative Medicine, Qingdao-Europe Advanced Institute for Life Sciences, BGI- Qingdao, Qingdao 266555, China; Lars Bolund Institute of Regenerative Medicine, Qingdao-Europe Advanced Institute for Life Sciences, BGI- Qingdao, Qingdao 266555, China; GigaScience Press, Norwalk, CT 06851, USA

## Abstract

Over the last decade, women have made decisive advances in increasing equality in science, technology, engineering, and medicine (STEM), but they still do not rival that of men. Many mechanisms to reduce gender discrimination have been addressed; however, little to nothing has been done to tackle the differences in the amount of time women spend on responsibilities at home. This has never been more apparent than during the COVID-19 pandemic. After a decade of advances promoting women, the last two years have seen these advances halted, and the long-term implications for women in STEM will be substantial. Moving forward, career advancement and funding mechanisms need to be adjusted to not just help women catch up, but to become a permanent support mechanism for women in the workplace. The higher amount of responsibilities at home and lack of support for women is not reserved for times of international upheaval: it has just become more apparent.

## Background

Gender imbalance has existed in the STEM fields for decades, and despite government and institutional efforts and resources devoted to addressing these issues, equality for female scientists in STEM remains elusive. While most of the early theories for gender stereotypes have been rejected, where there was a focus on physiological differences between men and women, stating that men and women have different ways of thinking, have different visual and spatial senses owing to anatomical differences, and that these fundamental limitations kept women out of science. Happily, most of those decades-old views of women being mentally or physically unfit to be involved in scientific activities have been dismissed. In fact, studies now show that rather than being unsuitable for research-related work, women often play a more important role in innovation and discovery (1). This finding, although showing the clear value of women for STEM fields, is likely due to women needing to accomplish more than men to have similar career trajectories. According to Bas Hofstra et al., who tracked the career path of over one million US students across all academic subjects from 1982 to 2010, there is a stratified system in R&D in which women must innovate at a greater level to obtain the same leadership rank as males (1).

Harassment in the workplace still plays a major role in discouraging women from staying in STEM fields. An assessment of the amount of workplace harassment has indicated that this has only been marginally addressed, with nearly 50% of women researchers reporting they have been the subject of workplace harassment over the last decade (2). Major changes in preventing harassment have been driven primarily by pressure from staff, male and female, and by external public outcry, making institutions take these issues more seriously.

Over the last decade, however, even with these institutional challenges, female scientists have overcome a large amount of gender bias in recruitment, promotions, publications, salaries, and amount of funding, among other areas (3). In addition with colleague and public pressure for change, there has been a growth of resources that are available to increase women's engagement in science and to stay in the workplace. A major one of these is the grass roots 500 Women Scientists (https://500womenscientists.org/) that have developed myriad resources that significantly aid in the development of long-term female networking resources, provide tools to foster long-lasting mentorship relationships, and provide career building resources for early career investigators. One important system has been their creation of a database to help institutions, conferences, and organizations “find” often overlooked women researchers: the “Request a Woman Scientist” database (4), which collects information about women researchers from around the world using a multidimensional questionnaire that is statistically and visually analyzed, and makes it easy for people to identify the “right woman for the job.”

There remains a need to move away from the women-in-science workshops and panels, which typically provide -in an hour or two- stale decades old advice: get a mentor and promote yourself, without any extensive detail on what to look for or aid in how to do this. (We'll just ignore the oft-mentioned recommendation for women to just “work hard” to succeed…) Platforms such as all-day (or longer) Women in Science Conferences can provide much more detail, advice, and activities to help women researchers with their careers. An example of this type of conference is the annual Women in Science Conference (http://gigasciencejournal.com/blog/women-in-science-2021/), now in its 8th year, run by the *GigaScience* editors and female researchers at BGI. At this conference, all the speakers and attendees are women. The talks combine scientific presentations, information on how senior women researchers have overcome difficulties they have faced due to their gender, as well as training in areas known to improve the trajectory of women's careers, such as how to write grants, as obtaining grants early in their careers makes a woman's career trajectory similar to that of their male colleagues. There is also a session where graduate students and postdocs present their work, often for the first time.

However, with all these improvements, one area that has been completely unaddressed, and one that female researchers in STEM continue to confront, is the lack of aid for women's responsibilities outside of work. These responsibilities include women typically bearing the largest proportion of work related to child and elderly care and household responsibilities. These activities, and the lack of support by institutions and failing government aid programs are key contributors to younger women leaving STEM fields as loss of early career support has a long-term negative impact on career trajectories. A survey conducted by the University of California, San Diego demonstrates how childbirth has differentially impacted women and men in the STEM field over the last eight years, showing that 43% of women left full-time jobs after having their first child, twice as many as new fathers (5). Responsibilities for household labor reduce working hours for both men and women; however, women tend to carry more of the burden of household labor in their families than their male counterparts: women spend 8.5 hours more per week on childcare and housework than men, and women in single-parent households spend twice as much time. This amount of time is significantly higher for single mothers where the cost of, or even availability of, childcare is lacking.

This gender disparity, and lack of support, in family care and household responsibilities has never been made clearer than the enormous negative impact the coronavirus pandemic has had on women with closures of nurseries and schools, and dangers to elderly family members. Multiple studies (e.g., 6) indicate that during the pandemic, women spent more time on childcare and home schooling than did men. For the majority of single mothers, this situation was far worse, especially financially (7). In the sciences, the most recent data indicate that the pandemic had a detrimental effect on publishing for both men and women; but there was a greater decrease in the number of manuscripts submitted by women and the number of articles published by women as first or corresponding authors. This correlated with the time spent working from home. The proportion of female authors increased following the end of home working (8). Emil Madsen et al. analyzed individual-level panel data from 431,207 authors in four biomedical-related fields and discovered that, while the epidemic had a negative effect on both male and female annual research output, women were disproportionately impacted, increasing the gender gap in research output. Women's production rates were 17% lower than men's in 2019 and 24% lower in 2020, with the biggest impact on women working in the early and middle phases of the research (9): a time when young women researchers need to do more than men to achieve similar career trajectories to men.

For STEM academics, the impact of lost time in the laboratory during the pandemic will have a long-term impact on career advancement for women, especially since much of career advancement and funding decisions in STEM are measured by the number of publications, or other specific evidence of progress in their research. Unless institutions and funding agencies changes assessment measures for career advancement and monetary support once life is normalized, there will be a complete loss of competitiveness for women for future advancement. It is critical, therefore, for universities and institutions to recognize the negative impact of a pandemic on female scientists, especially for women early in their careers, where their scientific productivity has the greatest effect, and to take steps to counteract this damage in order to protect female scientists.

## Conclusions

The disproportionate impact of the pandemic has shown quite clearly, that despite other mechanisms that universities and institutions have put in place promote fairness and enhance women's engagement in STEM, there is a clear need to set up mechanisms to mitigate the consequences of loss of professional work time due to their greater burden work in the home during the pandemic—but this area must become standard given that, even in “normal” times, these responsibilities disproportionately impact women. While some things have been done to reduce the effect of family and home care, many of these are limited and, more, unsuccessful, for continuing to support women in the workplace (10).

## Editor's Note

This commentary is part of a series to celebrate a Decade of *GigaScience*, to coincide with the 10th anniversary of our launch in July 2012. These papers take a look back at 10 years of advances in large-scale research as open science has become mainstream, and ways to increase opportunities for researchers in multiple areas. Since its launch, *GigaScience* has promoted increased learning opportunities for women in science by running conferences geared to aid young women in STEM, providing sponsorship for women to attend conferences, and covering costs for services (such as childcare services) that are especially useful for women at conferences.

## Competing Interests

The authors declare they have no competing interests.
